# Prion Protein: The Molecule of Many Forms and Faces

**DOI:** 10.3390/ijms23031232

**Published:** 2022-01-22

**Authors:** Valerija Kovač, Vladka Čurin Šerbec

**Affiliations:** Centre for Immunology and Development, Blood Transfusion Centre of Slovenia, Šlajmerjeva 6, SI-1000 Ljubljana, Slovenia; valerija.kovac@ztm.si

**Keywords:** prion protein, prion protein fragments, neuroprotection, myelination, ischemic stroke, neurodegenerative disease

## Abstract

Cellular prion protein (PrP^C^) is a glycosylphosphatidylinositol (GPI)-anchored protein most abundantly found in the outer membrane of neurons. Due to structural characteristics (a flexible tail and structured core), PrP^C^ interacts with a wide range of partners. Although PrP^C^ has been proposed to be involved in many physiological functions, only peripheral nerve myelination homeostasis has been confirmed as a bona fide function thus far. PrP^C^ misfolding causes prion diseases and PrP^C^ has been shown to mediate β-rich oligomer-induced neurotoxicity in Alzheimer’s and Parkinson’s disease as well as neuroprotection in ischemia. Upon proteolytic cleavage, PrP^C^ is transformed into released and attached forms of PrP that can, depending on the contained structural characteristics of PrP^C^, display protective or toxic properties. In this review, we will outline prion protein and prion protein fragment properties as well as overview their involvement with interacting partners and signal pathways in myelination, neuroprotection and neurodegenerative diseases.

## 1. Introduction

Prion protein (PrP) is a highly conserved ubiquitous glycoprotein. It exists in two forms; the normal or cellular isoform, PrP^C^, and the disease-associated infectious isoform or scrapie PrP, PrP^Sc^. The pathological role of PrP^Sc^ has been extensively studied in prion disease and has been reviewed in several papers [1,2,3]. PrP^C^ is expressed in a variety of different organs and tissues with high expression levels in the central and peripheral nervous systems. It is abundantly present on the cell surface of neurons [4,5,6] and has been shown to be involved in many physiological mechanisms. The function of the protein remains to be elucidated; nevertheless, intensive studies link PrP^C^ to myelin homeostasis [7], neuroprotection [8,9], the circadian rhythm [10,11], metal ion homeostasis [12,13], mitochondrial homeostasis [14] and intercellular signaling [6,15,16]. In neurons, PrP^C^ is present in the presynaptic and postsynaptic compartments of axon terminals where it is involved in anterograde and retrograde axonal transport [17,18,19,20]. PrP^C^ is cleaved at the cell membrane by proteases, forming released and attached forms. In recent years, prion protein and prion protein released forms have received attention in correlation with neuroprotection in neurodegenerative diseases. In this review, we present prion protein and prion protein released forms, summarize their involvement in myelination, neuroprotection and neurodegenerative diseases and discuss the most recent discoveries in this field.

## 2. Prion Protein

**Figure 1:**
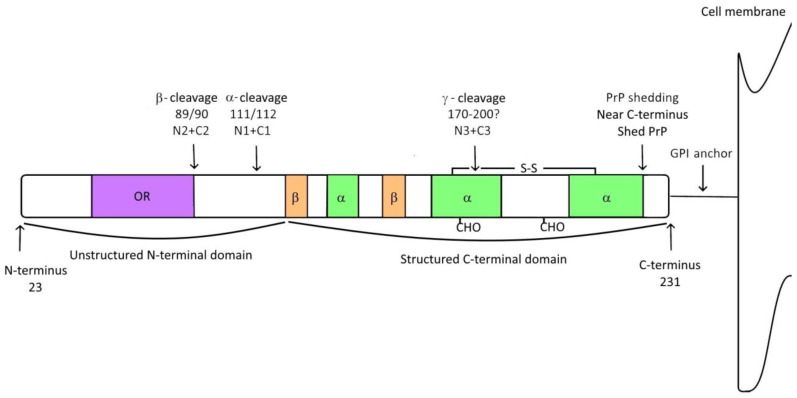
Schematic presentation of PrP^C^ with associated cleavages. Mature PrP^C^ is approximately 210 amino acids long. The flexible unstructured N-terminal part (residues 23–120) contains the octapeptide repeat region (OR, purple) whereas the highly structured C-terminal part (residues 121–231) is composed of three α-helices (green), two β-sheets (orange), a disulfide bond, two N-glycans (CHO; positions 181 and 197) and a C-terminal GPI anchor. PrP can undergo four cleavages: α-cleavage (cleavage site position 111/112); β-cleavage (cleavage site position 89/90); γ-cleavage (cleavage site presumably between positions 170–120); and shedding (near the C-terminus of PrP). Cleavages result in released (N1, N2, N3, shed PrP) and attached (C1, C2, C3) fragments of PrP^C^.

Mature human PrP^C^ is composed of a flexible unstructured N-terminal domain (amino acid residues 23–120) and a structured C-terminal domain (amino acid residues 121–231). It is anchored to the cell membrane with a glycosylphosphatidylinositol (GPI) anchor [21,22]. The flexible N-terminal domain contains an octarepeat region whereas the structured domain consists of three α-helices, two β-sheets, a disulfide bond connecting cysteines 179 and 214 and two N-glycans on amino acid residues 181 and 197 [23,24] (Figure 1).

PrP^C^ can transform into a β-sheet-rich isoform PrP^Sc^, which is prone to autocatalytic conversion and aggregation into insoluble aggregates [22,25,26]. An abnormal accumulation of the pathologic protein in the brain can cause the development of transmissible spongiform encephalopathies (TSEs), also known as prion diseases. Prion diseases include Creutzfeldt–Jakob disease (CJD), Gerstmann–Sträussler–Scheinker syndrome (GSS), fatal familial insomnia (FFI) and kuru in humans, bovine spongiform encephalopathy in cattle, scrapie in goats and sheep and chronic wasting disease in cervids. All prion diseases are rare fatal neurodegenerative disorders. The clinical and neuropathological features of prion diseases in humans are similar to those of Alzheimer’s disease (AD) such as rapid memory loss and loss of brain function as well as dementia, spongiform deformation of the brain, personality changes and difficulties with movement [15,27]. Although prion diseases occur due to the accumulation of toxic PrP^Sc^ aggregates in the brain, the mechanism that underlies the conversion of PrP^C^ to PrP^Sc^ and the development of prion disease remains an unknown. Apart from being a substrate for the development of prion diseases, PrP^C^ can serve as a receptor for cytotoxic amyloid-β (Aβ) oligomers [20,28] and toxic soluble aggregates of tau protein in AD and other tauopathies [29,30]. There are also opposing studies on PrP^C^ binding of α-synuclein (α-syn) oligomers in Parkinson’s disease (PD) and other synucleinopathies, opening the debate on the role of PrP^C^ in toxicity of α-synuclein [30,31,32,33].

## 3. Prion Protein Fragments

PrP^C^ can undergo four posttranslational cleavages, forming PrP fragments (Figure 1). The α-cleavage and β-cleavage occur within the unstructured N-terminal domain whereas the γ-cleavage and PrP shedding occur within the structured C-terminal domain. Apart from the mentioned cleavages, PrP^C^ has been cleaved under experimental conditions with phospholipase C, which cleaved PrP^C^ within the GPI anchor [34,35]. The site of cleavage, length of fragment and membrane attachment allow fragments to take part in various mechanisms.

### 3.1. α-Cleavage

The α-cleavage is the most studied cleavage of PrP^C^. It occurs under physiological conditions in the central hydrophobic region of mature PrP^C^ (amino acid residues 105–120 in human sequence 111/112) [36,37,38] (Figure 1). The cleavage releases an ~11 kDa fragment N1 whereas the ~18 kDa part C1 remains attached to the cell membrane by the GPI anchor [36,39]. For now, there is no unique enzyme responsible for the α-cleavage [24,40]. Although cleavage sites have been determined with respect to species, the α-cleavage is tolerant to sequence variation in this region as long as its hydrophobicity remains preserved [38]. Studies have shown that α-cleavage in the human brain, mouse models and neuronal cultures occurs in the presence of enzymes ADAM10 and ADAM17 [41,42,43]. ADAM10 contributes to a constitutive N1 production whereas ADAM17 mainly participates in N1 formation upon stimulation [44,45]. ADAM8 has also been shown to cleave PrP^C^ to form N1 and C1 in muscles [46]. A role of ADAM8, ADAM10 and ADAM17 in the α-cleavage has also been supported in a biophysical study [47]. Fragment N1 has a relatively low stability; nevertheless, it was found to be present in body fluids, tissue homogenates or cell culture supernatants [39,48,49]. The cleavage was initially thought to take place in acidic endosomal compartments [50,51] but later studies demonstrated that the α-cleavage occurs during the vesicular trafficking of PrP^C^ along the secretory pathway [52,53]. The α-cleavage uses PrP^C^ as a substrate, leading to its reduction of the cell surface. As PrP^C^ is also a substrate for prion replication and a key mediator of toxicity in prion diseases, AD and other neurodegenerative diseases, the cleavage has a positive biological effect. The flexible N-terminal part of PrP^C^ is essential for the interaction of the protein with the binding partners that regulate PrP^C^ uptake in trafficking [54,55]. Lacking N1, C1 forms complexes on the cell membrane [56] and is more stable and persistent at the cell surface than PrP^C^ [50]. Fragment C1 can be cleaved at the cell surface and released into the extracellular space [57]. C1 was found to inhibit prion replication in mice [58,59] whereas fragment N1 is neuroprotective [60,61]; the absence of the α-cleavage is toxic for both cells and mice [47,62].

### 3.2. β-Cleavage

The β-cleavage takes place at the end of the octapeptide repeat region N-terminal of the α-cleavage site. The β-cleavage is mostly observed under pathological conditions and is similar to the α-cleavage. It seems to act protectively. It takes place around amino acid residue 90, forming fragment N2 (~9 kDa) and fragment C2 (~20 kDa) [36,37,48,63] (Figure 1). The β-cleavage of PrP^C^ is mediated by reactive oxygen species (ROS) [37,63,64,65,66]. By removing ROS, the cleavage protects cells from oxidative stress [65]. Apart from ROS, the β-cleavage is induced by calpains [67], lysosomal proteases [68,69] or even ADAM8 [47]. Proteinase K cleaves the protease-resistant core of PrP^Sc^ (PrP27–30) near position 90, creating a fragment with a length similar to C2. Similar to fragment C1, fragment C2 can also be shed from the cell surface [70]. The formation of such a fragment indicates that proteases involved in the β-cleavage could also be involved in the cellular attempts to break down PrP^Sc^ [71,72].

### 3.3. γ-Cleavage

The most recently discovered protease cleavage of PrP^C^ is the γ-cleavage. The cleavage site in PrP^C^ remains to be determined but the sizes of the released fragment N3 (~20 kDa) and GPI-anchored fragment C3 (~5 kDa) suggest that protein cleavage occurs in the region between amino acid residues 170 and 200 [73,74] (Figure 1). Studies indicate that the γ-cleavage occurs late in the secretory pathway on an unglycosylated protein in the presence of members of the matrix metalloproteases (MMP) family [73]. The reason the γ-cleavage occurs only on unglycosylated PrP^C^ is proposed to be due to the steric hindrance of proteases by glycans in the proximity of the proposed cleavage site [40,75]. The γ-cleavage has been found to exist in different species, tissues and cell culture models. The determination of its role requires further study although an indication of increased amounts of fragment C3 in a CJD brain may lead to a possible pathogenic significance [73].

### 3.4. Shedding of Prion Protein

There is also an important cleavage of PrP in proximity to the C-terminus. The cleavage sheds PrP into the extracellular space, leaving a small number of amino acid residues on the cell surface. The cleavage was described in early research [35,39,76,77] but has received more attention in recent years due to the involvement of shed PrP in diseases [40,63,78,79,80,81,82,83]. Similar to the α-cleavage, the shedding of PrP occurs in the presence of enzymes from the ADAM family. In vitro and in vivo experiments suggest that ADAM9 and ADAM10 are involved in the process of cleavage and the shedding of PrP [47,84,85,86] where ADAM10 is the primary sheddase for PrP and ADAM9 is the modulator of ADAM10 activity [24]. Shed PrP was first determined in hamsters. In the prion-infected brain of hamsters, shed PrP represented approximately 15% of the PrP^Sc^ molecules [76]. A further analysis showed that ADAM10 cleaved shed PrP between Gly228 and Arg229 and formed shed PrP that terminated at Gly228 [84]. An analysis exploring the cleavage site profile of ADAM10 revealed that cleavage is not induced by a unique sequence [87]. Consequently, the ADAM10 protease can produce variants of shed PrP depending on the protein sequence and conformation. Jansen and coworkers described the existence of unanchored PrP forms ending with Tyr225 and Tyr226 in patients with prion disease [88]. The authors characterized two patients with prion disease who carried stop mutations at positions Y226X and Q227X and expressed the respective forms. Using a monoclonal antibody V5B2 [89] that specifically binds to a fragment of PrP ending with Tyr226, we concurrently described the existence of a free form of PrP named PrP226* [90,91,92,93,94]. The distribution of PrP226* in the human brain has been associated with the distribution of PrP^Sc^ [90,94]. Due to the existence of more than one shed form, we hypothesized that the proteolytic site in the human sequence is not exclusively located between amino acid residues 228 and 229 but is located in the proximity of the C-terminus [95] (Figure 1). Recently, Linsenmeier et al. published a comprehensive study on the mechanism stimulating PrP^C^ proteolytic shedding [81]. Using animal models and controls, they showed that PrP shedding negatively correlates with prion conversion and that shed PrP is abundantly present in amyloid plaques. They also studied the influence of the binding of PrP-directed antibodies to PrP^C^ in relation to shedding propensity. The binding of whole anti-PrP antibodies to the C-terminal structured domain of PrP^C^ or single-chain antibody derivatives, directed towards repetitive epitopes within the octarepeat region of the N-terminal domain stimulated shedding, when the binding of whole anti-PrP antibodies to the octarepeat region of the N-terminal domain locked the N-terminal domain structure and evoked PrP^C^ surface clustering, endocytosis and degradation in lysosomes [81].

## 4. Prion Protein and Myelination

PrP^C^ is abundantly expressed in the central nervous system (CNS) and in the peripheral nervous system [4,5]. Studies in primate brains, rodent brains and transgenic mice showed that it is enriched along axons and in presynaptic terminals where it is involved in anterograde and retrograde axonal transport [4,17,18,96,97,98]. Deletions in the PrP^C^ α-cleavage region showed severe demyelination in both the spinal cord and cerebellar white matter in vivo [99,100] Later, it was confirmed that axonal PrP^C^ and its α-cleavage are necessary for pro-myelination in the peripheral nervous system [101]. Using a co-isogenic PrP-knockout mice model, Kuffer et al. discovered that axonal PrP^C^ promotes myelin maintenance in trans via binding to the adhesion G-protein-coupled receptor Adgrg6 on Schwann cells with an N-terminal flexible tail [7]. They also confirmed that mice lacking PrP^C^ developed chronic demyelinating neuropathy, which suggests that myelination homeostasis in the peripheral nervous system is a bona fide physiological function of PrP^C^ [7]. Myelin maintenance was found to be regulated through the binding of an N-terminal released fragment of PrP^C^ (presumably N1 or shed PrP) to Adgrg6 on Schwann cells. The interaction activated Adgrg6, increased the cellular levels of cAMP and triggered a signaling cascade that promoted myelination [7]. The regulation of peripheral myelin maintenance by PrP^C^ was confirmed in five different PrP-knockout mouse model strains that developed late-onset peripheral neuropathy [101,102,103]. Recently, there was an attempt to develop a treatment for peripheral demyelinating diseases based on binding between the N-terminal domain of PrP^C^ and Adgrg6 [104]. In this study, they constructed an immunoadhesin molecule consisting of two flexible N-terminal domains of PrP^C^ linked to a crystallizable fragment (Fc) of immunoglobulin G1 (FT_2_Fc) [104]. The molecule showed favorable pharmacokinetic properties and showed potential in vitro but failed to have a therapeutic effect on the early molecular signs of demyelination in PrP-knockout mice [104]. PrP^C^ was also studied in connection to peripheral myelin development and regeneration after nerve injuries [105]. As PrP was found to be dispensable in this mechanism, it could be presumed that PrP has no major role in the peripheral nerve repair process or its absence might be compensated by other ligands [105].

Myelination and other physiological roles of PrP^C^ have been intensively studied on animal models with a knocked-out or knocked-down PrP gene expression. Studies have shown limited negative effects in mice [102,106,107,108,109], cattle [110] and goats [68,111,112] whereas studies on PrP-knockout mice or goats showed defects in the nervous system and sensitivity to oxidative stress [6,101,111,113]. Several PrP-knockout mice models were generated with a mixed background [106,109,114,115,116]. As the studies are not reproducible among models, this might raise the question of whether any observed phenotypes were actually due to polymorphisms in genes flanking *Prnp* or the result of PrP^C^ absence. To avoid this issue, it would be advisable to repeat key experiments using co-isogenic PrP-knockout mice.

Although the role of PrP^C^ in the CNS needs to be elucidated, PrP^C^ and PrP^C^ released fragments are indispensable in peripheral nerve myelin homeostasis but they may be dispensable in nerve recovery.

## 5. Prion Protein and Ischemic Strokes

In the previous section, we observed that knockout animals are more vulnerable to oxidative stress. Studies support the idea that PrP^C^ acts as an antioxidant by regulating glutathione reductase activity [117,118] and by regulating superoxide dismutase (SOD) through ion binding [119,120,121,122,123]. PrP-knockout mice showed a reduced protection against ROS whereas prion-infected mice showed increased levels of oxidative stress, most likely as a consequence of a PrP^C^ loss of function [124,125,126]. Under oxidative stress conditions, PrP mRNA levels increase, which implies that oxidative stress upregulates PrP^C^ expression [127]. Ischemic stroke is a condition where the loss of blood flow in a brain area causes hypoxic conditions and brain damage [128]. PrP-knockout animal models subject to ischemia showed intensive ischemic damage and a reduced chance of regeneration whereas the possibility of PrP^C^ synthesis resulted in PrP^C^ overexpression and decreased ischemic damage [127]. Studies on ischemic strokes have indicated that PrP^C^ overexpression can reduce the lesion size compared with wild-type mice, ascribing PrP^C^ a protective role in ischemia damage [129,130,131,132,133,134,135]. After an ischemic insult, PrP^C^ is associated with neuroprotective and regenerative processes by interacting with various cytosolic and transmembrane signal proteins. Among others, PrP^C^ has been associated with the upregulation of extracellular signal-regulated kinase (ERK1/2) [133,136,137], activation of the phosphatidylinositol 3-kinase/protein kinase B/Akt (PI3K/Akt) pathway [138,139,140,141,142], modulation of N-methyl-D-aspartate (NMDA) receptor-mediated toxicity [143], activation of the cAMP-dependent protein kinase A (PKA) pathway [144,145,146] and interaction with stress-inducible protein 1 (STI1) [146], all resulting in neuron survival, neurite outgrowth and neuroprotection.

PrP^C^ is a receptor of Fyn kinase, a member of the Src family of tyrosine kinases (SFKs) [146]. Through Fyn kinase activation, PrP^C^ mediates oligomer-induced toxicity in neurodegenerative diseases [147,148,149,150] and promotes neurite outgrowth by the phosphorylation of the GluN2A domain of the neuronal cell adhesion molecule (NCAM) [151]. Fyn kinase and other members of the SFK family are involved in ischemic damage [152,153,154,155]. The inhibition of SFKs in a global ischemia model and the inhibition of the Fyn-mediated phosphorylation of GluN2A in a model of neonatal HII resulted in an increased neuronal survival [156,157,158] whereas the overexpression of Fyn in the model of neonatal HII led to increased brain damage [159]. The inhibition of SFKs in a mouse model of an ischemia also resulted in a decreased ischemic volume and improved cerebral function after provocation [155]. As this effect was not seen in Fyn-knockout mice, we suspect that ligands other than Fyn kinase may also affect ischemia insult recovery [155].

PrP^C^ fragments were also shown to be involved in ischemic stroke. Fragments N1 and N2 were shown to act protectively under cellular stress [160,161,162] and modulate the quiescence of neural stem cells in adult neurogenesis upon stroke [163] whereas PrP^C^ fragments C1 and C2 were involved in regulating p53-dependent apoptosis and cell survival [164]. Fragment C1 was found to be enriched in small EVs (sEVs) where it acted similarly to viral surface proteins [165,166]. Due to this, it may affect the intercellular information exchange between sEVs and their target cells as well as contributing to their uptake [63]. Brenna et al. studied the similarities between the cellular uptake of brain-derived sEVs from PrP-knockout mice and wild-type mice after a stroke [128]. They showed that sEVs lacking PrP were taken up significantly faster with a greater efficiency and were more easily sorted into lysosomes than sEVs containing PrP and fragment C1 [128]. Fragment N1 was also found to be involved in regulating the interactions between microglia and other brain cells. A recent in vitro study on a mixed neuronal lineage and microglia co-culture system showed that fragment N1 stimulated a change in the cell morphology and metabolism and induced Cxcl10 secretion [167]. Furthermore, fragment N1 was shown to influence microglia to change the membrane composition to a higher GM1 content at the interaction sites with the surrounding cells in a co-culture yet only upon direct cell-to-cell contact [167]. Fragment N1 was also proposed to protect neurons against staurosporine-induced Caspase-3 activation in an ischemic model of the rat retina [60]. These results are supported by in vitro studies where the expression of PrP^C^ was protective against staurosporine or anisomycin-induced apoptosis [144,146]. Fragment N1 is also related to neuroprotection in neurodegenerative diseases, which is discussed in more detail in the next section. In the presence of anchored PrP^C^, recombinant PrP (recPrP) can induce ERK1/2 and Akt signaling on mesenchymal stem cells that may support neuronal differentiation [168], promote neurite outgrowth and facilitate axonal growth cone guidance [169]. Recently, it was reported that recPrP promotes neurite outgrowth and Schwann cell migration through the ERK1/2 pathway [170]. The activation involved NMDA receptors, low density lipoprotein receptor-related protein-1 (LRP1), SFKs and Trk receptors; it seemed to take place independently of anchored PrP^C^ [170]. In this mechanism, SFKs played a critical role in recPrP-initiated cell signaling by activating Trk receptors, which are upstream of ERK1/2 [170,171]. Although recPrP lacks glycosylation, it might be considered to be a suitable analog of shed PrP.

Prion protein and prion protein fragments are linked with intercellular communication and signaling, oxidative stress and neuroprotection and present an attractive target for the treatment and regulation of these mechanisms. Nevertheless, further studies should be conducted to confirm the effects of these molecules in the mentioned mechanisms.

## 6. Prion Protein and Neurodegeneration

Neurodegeneration is the progressive loss of the structure or function of neurons, which may ultimately involve cell death. On the molecular level, neurodegeneration is connected to accumulation of misfolded proteins. Accumulation of protein aggregates causes mitochondria dysfunction, induces oxidative stress and ultimately causes chronic inflammation. Neurodegeneration occurs in diseases such as prion disease, PD and AD due to the aggregation of PrP^Sc^ [26,172,173], α-syn [174,175,176,177] and Aβ isoforms [178,179] and tau protein [180,181,182,183], respectively. Prion protein or prion protein fragments have been found to interact with aggregating agents in different neurodegenerative diseases but their roles depend on the studied conditions [24,81,184,185].

It has been reported that PrP^C^ binds a wide range of β-sheet-rich oligomers associated with neurodegenerative diseases [148,149,150]. PrP^C^ engages metabotropic glutamate receptor 5 (mGluR5) and mediates oligomer-induced toxicity through Fyn kinase [175,186,187,188]. Activated Fyn kinase can phosphorylate the GluN2A and GluN2B subunits of NMDA receptors, which are then hyperactivated and cause calcium influx and cell death [20,189]. It has also been shown that PrP^C^ can activate Fyn kinase-mediated Aβ oligomer toxicity by an interaction with LRP1 [190]. A recent study in this field suggested that, apart from LRP1, this process includes activated a2-macroglobulin and tissue-type plasminogen activator [191]. Studies have implied that binding between soluble protein aggregates and PrP^C^ causes neurotoxicity and inhibits long-term potentiation (LTP) [30,192]. Opposing studies have also been published that report no significant effect of PrP^C^ levels on Aβ-induced LTP in PrP-knockout mice [193], cell ablation or PrP overexpression [194]. The reasons for these discrepancies are unclear but they could be due to the use of different model systems and toxic or nontoxic species [195].

Aβ oligomers bind to PrP^C^ at two binding sites within the flexible N-terminal part of PrP^C^, between amino acid residues 23–27 and 92–110 [192,195,196]. Apart from Aβ oligomers, PrP^C^ has been reported to be a receptor for α-syn oligomers and tau aggregates. Similar to Aβ oligomers, anchored PrP^C^ binds small soluble aggregates or shorter fibrils of α-syn oligomers or tau aggregates within the flexible N-terminal part [30,175,185,197,198,199]. PrP^C^ has also been shown to uptake recombinant α-syn fibrils. A model system lacking PrP^C^ showed a lower uptake of α-syn and α-syn fibrils in comparison with controls [177,185,197], resulting in less α-syn aggregation, astroglial activation and loss of dopaminergic neurons in the brains of PrP-knockout mice [185]. Furthermore, PrP-knockout mice did not exhibit α-syn-induced LTP impairment whereas treatment with an anti-PrP antibody prevented α-syn-induced LTP defects in a model of PD [175]. Although the mentioned studies support a PrP^C^ and α-syn oligomer interplay, La Vitola et al. showed that PrP^C^ was not mandatory for the mediation of α-syn oligomer detrimental effects in vitro or in vivo [33]. Although the discrepancy could not be explained in the study, it could also occur due to the use of a different protocol of soluble aggregate preparation or the use of different model systems. Anchored PrP^C^ was also shown to bind tau aggregates and seemed to facilitate their uptake [30,198,200]. Absence of PrP^C^ or pretreatment with anti-PrP blocking antibodies was shown to decrease the uptake of recombinant tau aggregates and abolish tau aggregate-induced toxicity [30,198,200].

Studies regarding recombinant PrP fragment N1 in neurodegenerative diseases have shown that these molecules can bind toxic Aβ oligomers at regions between amino acid residues 23–31 and 95–105. Fragment N1 neutralizes toxic Aβ oligomers by seizing them in the extracellular space and reduces oligomer-induced toxicity [61,195,201,202,203,204]. The protective effects of fragment N1 have also been observed in vivo in mice exposed to acute Aβ-induced toxicity [203]. Beland and coworkers observed increases in the α-cleavage of PrP^C^ in the brains of AD patients [205]. As the N1 fragment abundantly binds Aβ oligomers, it may be indicated that the cleavage acts protectively in the development of diseases [205] whereas the inhibition of N1 production promotes AD progression [42].

PrP shedding reduces the level of cell-anchored PrP^C^ [78]. This results in a decreased level of the substrate for prion replication and a decreased level of the receptor for toxic oligomers [85,206]. Similar to fragment N1, shed PrP is also believed to be protective in prion diseases and other neurodegenerative diseases [40,79,81]. As mentioned in the previous section, recPrP is similar to shed PrP. Although it lacks glycans, recPrP may be used as a model to predict the role of shed PrP in diseases. RecPrP was found to increase the development of synapses and neurite outgrowth in the presence of anchored PrP^C^ [170,207]. Similar to fragment N1, recPrP also inhibited Aβ oligomer formation and neutralized Aβ oligomer toxicity in an AD model [203]. In vitro studies using recPrP and its derivatives showed that both the N-terminal and C-terminal domains of PrP are required for an efficient inhibition of Aβ fibril elongation [202,208] and support the protective role of shed PrP in the inhibition of Aβ fibril formation. RecPrP was also shown to bind tau aggregates and α-syn oligomers and may neutralize their toxicity [30]. Although PrP^C^ shedding acts protectively, enhanced PrP^C^ shedding could lead to negative biological activity such as inflammation in the CNS [83,209]. Jarosz-Griffiths et al. [82] recently reported on the protective role of PrP shedding. The authors reported that siRNA-mediated ADAM10 knockdown reduced PrP^C^ shedding and increased Aβ oligomer binding whereas acitretin promoted PrP^C^ shedding and decreased Aβ oligomer binding in the neuroblastoma cells and in human-induced pluripotent stem cells [82].

In a recent paper by Linsenmeier et al., researchers evaluated the role of shed PrP in different models [81]. Using a polyclonal antibody sPrPG228 that specifically recognized murine PrP ending with G228 [210] they showed that in prion-diseased mice, shed PrP colocalized with PrP^Sc^ in amyloid plaques. Similar to the model of prion disease, shed PrP was also distributed to Aβ deposits in the brains of 5xFAD mice where it was found bound to Aβ oligomers and seen in the center of many amyloid plaques. Due to the knowledge in this field thus far, the authors proposed that physiologically shed PrP may act protectively in prion diseases and AD by blocking toxic oligomers and/or by precipitating them into less toxic deposits [81,211].

RecPrP and N1 may also inhibit Aβ oligomerization, neutralize cytotoxicity of preexisting Aβ oligomers, prevent the binding of oligomers with cell surface PrP^C^ and rescue the Aβ-induced impairment of LTP [212]. As recPrP and N1 both contain proposed binding sites of protein oligomers, both molecules were reported to also bind α-syn oligomers as well as mediate the co-clustering of α-syn oligomers and AD-associated amyloid-β oligomers [199].

PrP^C^ is enriched in extracellular vesicles (EVs) [128,213,214]. Little is known regarding the physiological functions of PrP^C^ in EVs. Several studies have suggested that PrP^C^ in EVs protect cells against Aβ toxicity [214,215,216,217]. The mechanism behind the neutralization of toxic Aβ oligomers by EVs is not known; nevertheless, it is presumed that it is similar to the recPrP or N1-mediated process. It has been proposed that exosomal PrP^C^ catches Aβ oligomers at the N-terminal PrP region (amino acid residues 23–31 and 95–105) [203], neutralizes the oligomers, promotes the formation of Aβ fibrils and upregulates internalization and degradation of the aggregates by microglia [214,215,216,217]. As recPrP and anchored PrP^C^ have been shown to bind tau and α-syn oligomers [30], exosomal PrPs are expected to act in the same manner. By binding free toxic tau or α-syn oligomers in the extracellular space, exosomal PrPs prevent toxic oligomer binding to anchored PrP^C^ and inhibit toxic signaling in the CNS of patients with diseases. Exosomes associated with PrP^Sc^ have been shown to be infectious and pose a danger of spreading prion disease [218,219,220,221,222]. Although there is no direct study yet, exosomal PrP^C^ might also induce CNS inflammation. More work needs to be undertaken to examine other biological activities that exosomal PrP^C^ may possess.

On the basis of the determined oligomer binding domains, researchers have designed potential treatment strategies for AD based on synthetic peptides [204,223] and functional Aβ oligomer-binding compounds [149]. The designed synthetic peptides have been shown to reduce the initial rate of Aβ fibrillization, inhibit the aggregation pathway of Aβ by reducing Aβ oligomer uptake and protect cultured hippocampal neurons from the oligomer-induced retraction of neurites and loss of cell membrane integrity [204] whereas D-peptide RD2D3 has been shown to be successful in interfering with the PrP^C^-Aβ oligomer assembly and has been proposed as a promising therapeutic agent in AD [223].

## 7. Conclusions

**Figure 2:**
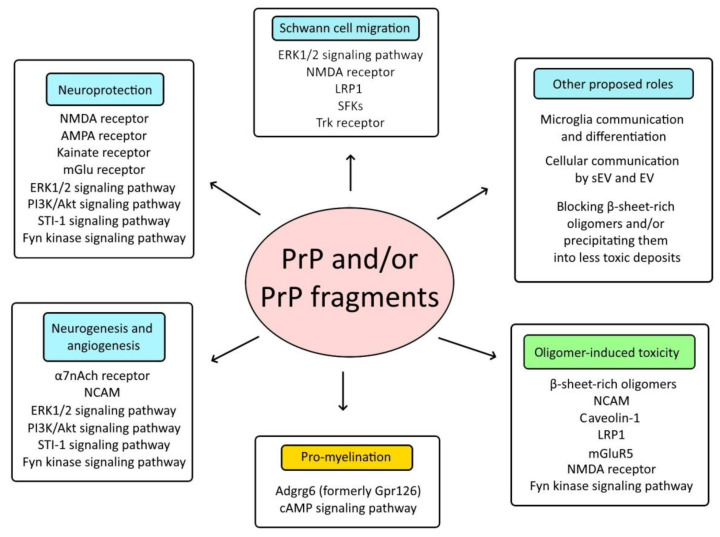
Proteins, signaling pathways and interactions that may be affected by PrP and/or PrP fragments. This scheme presents various proteins, signaling pathways and interactions that reportedly involve PrP and/or its fragments. In ischemic stroke, PrP species were found to be involved in modulating neuroprotection, neurite outgrowth, neurogenesis and angiogenesis. In neurodegenerative diseases, released PrP fragments may act protectively whereas anchored PrP regulates oligomer-induced toxicity. PrP and its derivatives are also involved in Adgrg6-induced myelination homeostasis (orange) and may be involved in microglia communication and differentiation as well as regulating intercellular communication through EVs and sEVs, etc. Several of the proposed interplays are regulated by a direct interaction with PrP species whereas others are regulated indirectly. Protective pathways and interactions are colored blue whereas green color presents harmful outcomes.

The reviewed studies support the fact that prion protein and/or prion protein fragments are involved in myelin homeostasis, ischemia and neurodegeneration where they may take on different roles (Figure 2). According to the current information, anchored PrP and/or released fragments (N1, shed PrP) interact with Adgrg6 to regulate peripheral nerve myelin homeostasis. Although there have been attempts to connect PrP to other Adgrg6-mediated processes, no direct involvement has been perceived. In strokes, the expression of PrP is upregulated. Anchored PrP takes part in mediating signaling pathways through transmembrane and cytosolic receptor proteins. Although further study is needed, the released forms may play decisive roles in neuroprotection and regeneration, including the regulation of interactions between microglia and brain cells and the promotion of neurogenesis. EVs and sEVs highly enriched in PrP fragments may be important delivery mechanisms in neuroprotection and neurodegeneration; further studies are needed to prove their roles. In neurodegenerative diseases, anchored PrP acts as a receptor for Aβ oligomers, α-syn oligomers and tau aggregates and may mediate oligomer-induced cytotoxicity. The point of interaction between the oligomer and PrP may be an attractive site for drug development but therapy may also include the regulation of other partners involved in this process. Arguing their protective role, released PrP fragments may bind toxic oligomers and enable their depletion. Supporting this role, shed PrP has been shown to bind PrP^Sc^ and Aβ oligomers in amyloid plaques, which may be less toxic than oligomers. To conclude, there are many indications suggesting that prion protein and prion protein fragments may have multiple (sometimes even intertwined) roles in strokes and neurodegeneration. To undoubtedly elucidate their role(s) in these processes, further studies are needed in these fields.
